# South Texas Medical Association

**Published:** 1897-05

**Authors:** E. S. Ferguson

**Affiliations:** Sec’y, 607½ Main St., Houston, Texas


### South Texas Medical Association.

DEAR DOCTOR:—The second semi-annual meeting of the South Texas Medical Association will meet in the Tremont Hotel, Galveston, May 14th, 1897.

The South Texas Medical Association is the representative society of this section of the State and should include all reputable physicians in South Texas. The fee is nominal ($1.00).

All extraneous matter is abolished, thus devoting all time to scientific discussion and research.

To increase its power and influence, we respectfully request your attendance and co-operation, and we desire your application for membership, and also, if convenient, to contribute some medical or surgical essay on any subject in which you may be interested.

Kindly notify us of the title as early as possible so that it may be entered on programme, which programme will be mailed about two weeks previous to meeting.

Yours truly,

E. S. Ferguson, M. D., Sec'y,

607½ Main St., Houston, Texas.

